# Gender-specification lifestyle factors associated with mild cognitive impairment among young-old adults in Taiwan

**DOI:** 10.18632/aging.206172

**Published:** 2024-12-10

**Authors:** Su-Wen Chuang, Ching-Wen Chen, Meng-Chang Lee, Yu-Hsuan Chen, Wen Su, Cheng-Jung Chen, Wei-Teing Chen, Po-Jen Hsiao, Chih-Chien Chiu, Sui-Lung Su

**Affiliations:** 1 Graduate Institute of Life Sciences, National Defense Medical Center, Taipei, Taiwan, ROC; 2 School of Public Health, National Defense Medical Center, Taipei, Taiwan, ROC; 3 Graduate Institute of Aerospace and Undersea Medicine, National Defense Medical Center, Taipei, Taiwan, ROC; 4 Division of General Surgery, Department of Surgery, Tri-Service General Hospital, National Defense Medical Center, Taipei, Taiwan, ROC; 5 Taichung Veterans General Hospital Chiayi Branch, Chiayi City, Taiwan, ROC; 6 Division of Thoracic Medicine, Department of Medicine, Cheng Hsin General Hospital, Taipei, Taiwan, ROC; 7 Department of Medicine, Tri-Service General Hospital, National Defense Medical Center, Taipei, ROC, Taiwan, ROC; 8 Division of Nephrology, Department of Medicine, Taoyuan Armed Forces General Hospital, Taoyuan, Taiwan, ROC; 9 Division of Nephrology, Department of Medicine, Tri-Service General Hospital, National Defense Medical Center, Taipei, Taiwan, ROC; 10 Division of Infectious Disease, Department of Internal Medicine, Taoyuan Armed Forces General Hospital, Taoyuan, Taiwan, ROC; 11 Division of Infectious Disease, Department of Internal Medicine, Tri-Service General Hospital, National, Defense Medical Center, Taipei, Taiwan, ROC

**Keywords:** young-old, mild cognitive impairment (MCI), MMSE, lifestyle, gender-specification

## Abstract

Background: The prevalence of mild cognitive impairment (MCI) exhibits a positive correlation with age, particularly evident in the old-old female population. Lifestyle factors have been identified as crucial risk determinants for MCI. However, there is a scarcity of research focusing on lifestyle factors among young-old population.

Objective: This study aimed to explore the lifestyle factors associated with MCI in young-old male and female.

Methods: This study employed a cross-sectional design and utilized demographic and lifestyle data obtained from participants enrolled in the Taiwan Biobank (TWB) between 2008 and 2021, with 32,897 individuals aged 60 to 70 years old. Cognitive function was assessed using the Mini-Mental State Examination (MMSE), with a total score ranging from 0 to 30 points. The cut-off of MCI scores was ≤18, ≤21, and ≤25 according to the education level, respectively. Logistic regression analysis was employed to assess the association between lifestyles and cognitive function.

Results: 3,878 individuals (11.78%) suffered from MCI. Upon gender stratification, high exercise metabolic equivalents in male (OR = 0.8, 95% CI: 0.70 - 0.92) and moderate exercise in female serve as protective factors for MCI (OR = 0.78, 95% CI: 0.70 - 0.87). Additionally, diversified dietary preferences among female (OR = 0.80, 95% CI: 0.66 - 0.97) also emerge as protective factors for cognitive function.

Conclusions: It is worth noting that male is advised to target a higher exercise metabolic equivalent, while female can attain cognitive benefits with moderate exercise and diversified dietary.

## INTRODUCTION

Aging has become a significant concern for most countries in recent years. In older adults, mild cognitive impairment (MCI) has been recognized to be one of the causes of elevated risk of mortality and morbidity globally. Besides, it is significantly taxing to the affected individuals, their caregivers, and society as a whole [1].

Given the absence of efficacious treatment options, early intervention is deemed the most cost-effective approach for dementia management [2]. As MCI is the transitional phase between uninjured cognitive function and dementia [3], it is beneficial to intervene in this phase, especially in young-old population, to prevent the onset of dementia [4].

While genetic inheritance is a significant risk factor for MCI or dementia, such as APOE ɛ4 and MTHFR [5, 6], some studies reveal that both lifestyle and genetic risk are independently linked to the risk of MCI [7]. Those findings show that an unhealthy lifestyle is associated with an increased risk of MCI, regardless of genetic factors [7, 8]. However, increasing research suggests that adopting a healthy lifestyle (such as not smoking, limiting alcohol consumption, maintaining a healthy weight, following a high-quality diet, and engaging in physical exercise) can reduce or delay the onset of cognitive impairment [9].

There is a plethora of research on the association between lifestyles and MCI in old-old population [10–12], but fewer studies have specifically focused on the young-old stage which may be a golden period to intervene and prevent. In studies conducted within the old-old population, an increased prevalence of MCI in females compared to males was observed, signifying a gender disparity [13]. Gaining insights into the gender-specific differences in young-old adults could contribute to a more profound comprehension of the etiology and preventive measures for dementia.

However, the effects of modifiable lifestyle factors on cognitive health have not been comprehensively investigated in Taiwanese young-old adults. It is necessary to systematically investigate and analyze this problem in a large community-based population. A prevention strategy should be developed to target the identified risk factors in the MCI population to decelerate disease progression. Therefore, this explores the role of different lifestyle factors in the prevention or development of MCI in the Taiwan Biobank (TWB) young-old male and female.

## MATERIALS AND METHODS

### Study design and population

This study data were derived from the TWB, an ongoing community-based prospective cohort study initiated in 2012, involving around 200,000 participants aged 20–70 years recruited from various regions across Taiwan. Following their informed consent to participate in the TWB study, participants undergo a physical examination and a structured questionnaire conducted by a well-trained researcher [14].

**Figure 1:**
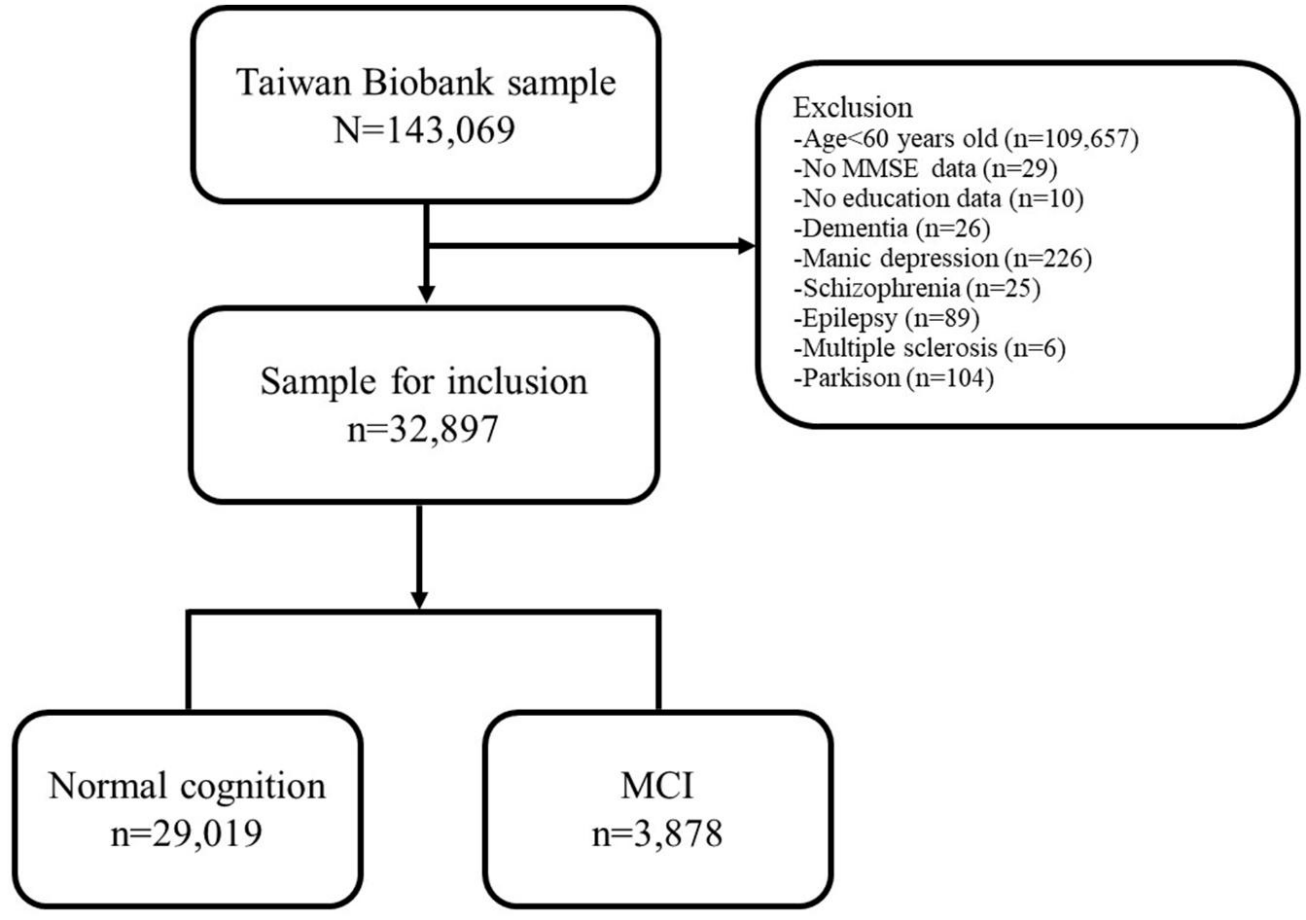
**Flow chart of the participants included in TWB.** Abbreviation: TWB, Taiwan Biobank; MCI, Mild Cognitive Impairment.

This study adopts a cross-sectional design, comprising structured questionnaire and physical examination of 143,069 participants from the TWB collected between December 2008 and May 2021. The research flow chart, as illustrated in Figure 1, involves excluding participants under the age of 60, those with missing data on MMSE (Mini-Mental State Examination) and education level, as well as individuals with mental disorders such as dementia, bipolar disorder, schizophrenia, epilepsy, multiple sclerosis, and Parkinson’s disease. Eventually, the study included 32,897 participants, who were divided into MCI (n = 3,878) and cognitively normal groups (n = 29,019) according to the MMSE score [15].

### Demographic factors

The demographic data includes gender, age, marital status, educational level, Body Mass Index (BMI), hypertension, hyperlipidemia, stroke, and diabetes.

### Lifestyle factors

In the lifestyle dataset, smoking was categorized as “yes” (including individuals who quit smoking less than 20 years ago) or “no” (including quitting more than 20 years). Alcohol consumption was defined as the consumption of 150 ml or more per week for at least 6 months, categorized as “yes” (including abstinence) or “no”. Tea consumption was defined as at least once a day, categorized as “yes” or “no”. Similarly, coffee consumption was defined as at least three times a week, categorized as “yes” or “no”. Supplement consumption was defined as the presence or absence of vitamins, minerals, or other supplements in the last month, categorized as “yes” or “no”.

The dietary patterns were derived from responses to 17 questions regarding food intake preferences using a five-point scale (1 = never, 2 = seldom, 3 = sometimes, 4 = frequently, and 5 = always). Dimension reduction was accomplished through factor analysis based on principal components analysis (PCA), and the components matrix shown in Supplementary Table 1. Several factors were determined according to the result of PCA. The interpretation of each factor was based on food categories with factor loadings greater than 0.5 after rotation. Ultimately, three dietary patterns were identified: meat-based, flavor-based, and fat-based dietary patterns. The “meat-based” factor represents preferences for lean meat, lower-fat content meat, and non-red meats. The “flavor-based” factor represents preferences for added seasonings, pickled vegetables, and fermented tofu, indicating a more diverse diet. The “fat-based” factor represents preferences for fried cooking methods and consumption of fatty meats and skin. Additionally, the scores for each dietary pattern were categorized into quartiles.

Exercise habits refer to asking participants whether they engage in regular physical activity, defined as exercising at least three times per week for more than 30 minutes per session. We used the most three common exercise types in the past three months self-reported by the participants to calculate the metabolic equivalent of task (MET), detailed in Supplementary Table 2 [16]. The accumulation of exercise activity was defined as the sum of MET per week, which was divided into none, ≤20 MET/week, and >20 MET/week. The exercise intensity levels were based on the Physical Activity Guidelines for Americans (PAG). According to PAG, participants were divided into three groups: group1: no exercise habit or only conduct light-intensity physical activity (<3 MET/hr); group2: conduct physical activities including moderate-intensity physical activity (3-5.9 MET/hr); and group3: conduct physical activity including vigorous-intensity physical activity (≥6 MET/hr) [17].

### Assessment of mild cognitive impairment

Cognitive impairment was assessed by the Mini-Mental State Examination (MMSE) according to the definition [18]. The evaluation items include orientation, attention, memory, language, oral comprehension and behavior ability, construction ability, and other items. The scores of MMSE range from 0 to 30, and higher scores indicate better cognitive function. The cut-off scores for MCI were defined as scores ≤18 for illiterates, ≤21 for participants with primary school education, and ≤25 for those with junior high school degree or above [15].

### Ethical issues and subjects

This study was authorized by the institutional review board of the Tri-Service General Hospital (TSGH-2-107-05-091). Written informed consent was not required for this study in accordance with national legislation and institutional requirements.

### Statistical analysis

For descriptive statistical methods, the study variables were presented in the following manners: (1) categorical (nominal) variables: the number of distributions and ratios; and (2) interval and ratio variables: average, and standard deviation. For inferential statistical methods, the single-variable analysis included Student’s t-test, and χ2 test to compare the demographic data and lifestyle data between cognitive normal and MCI participants, while logistic regression was used to adjust for possible confounding factors. This study considered a p-value of <0.05 as significance for all analyses. All analyses were performed using R 3.5.2.

### Availability of data and materials

The data supporting the findings of this study are available from Taiwan Biobank (https://www.twbiobank.org.tw). According to Taiwan Biobank’s policy, the availability of these data is restricted. These data are used under the license of the current study and therefore not public.

## RESULTS

### Demographic characteristics

The demographic characteristics of participants were summarized in Table 1. The total number of participants was 32,897, with 39.1% male and 60.9% female. The average age of the participants was 64.03 ± 2.96 years. In the cognitively normal group, which constituted 88.22% of the total population, the average age was 63.99 ± 2.94 years. In the group with MCI, which accounted for 11.78% of the total population, the average age was slightly higher at 64.31 ± 3.08 years. The MCI group was characterized by older age (p<0.001), lower proportion of married individuals (p<0.001), lower educational levels (p<0.001), and higher body mass index (BMI) (p<0.001) compared to that of normal group. Meanwhile, the MCI group also had a higher prevalence of hypertension (p<0.001), diabetes (p<0.001), and stroke (p<0.001).

**Table 1:** Demographic of participants with/without MCI.

**Variable**	**Total (n=32,897)**	**MMSE^a^**		**p-value**
		**Normal (n=29,019)**	**MCI (n=3,878)**	
Sex				0.594
Male	12,862 (39.1%)	11,361 (39.2%)	1,501 (38.7%)	
Female	20,035 (60.9%)	17,658 (60.8%)	2,377 (61.3%)	
Age (y)	64.03±2.96	63.99±2.94	64.31±3.08	<0.001
Marital status				<0.001
Married	25,488 (77.5%)	22,590 (77.9%)	2,898 (74.8%)	
Unmarried/Widow/Divorced	7,390 (22.5%)	6,414 (22.1%)	976 (25.2%)	
Educational levels				<0.001
Junior high school above (>6y)	27,890 (84.8%)	24,720 (85.2%)	3,170 (81.7%)	
Primary school (≤6y)	4,826 (14.7%)	4,196 (14.5%)	630 (16.2%)	
Illiteracy	181 (0.6%)	103 (0.4%)	78 (2.0%)	
Education				<0.001
≤12 y	19,136(58.2%)	16,152(55.7%)	2,984(76.9%)	
>12 y	13,761(41.8%)	12,867(44.3%)	894(23.1%)	
BMI (kg/m^2^)	24.36±3.38	24.31±3.37	24.74±3.46	<0.001
Hypertension	8,273 (25.1%)	7,175 (24.7%)	1,098 (28.3%)	<0.001
Diabetes	3,621 (11.0%)	3,117 (10.7%)	504 (13.0%)	<0.001
Stroke	447 (1.4%)	367 (1.3%)	80 (2.1%)	<0.001
Hyperlipidemia	4,823 (14.7%)	4,299 (14.8%)	524 (13.5%)	0.031

^a^MCI was defined as a score ≤18 for illiterates, ≤21 for participants with primary school education, and ≤25 for those with junior high school degree or above.

### Lifestyle characteristics

The lifestyle characteristics of participants are presented in Table 2. Compared to the normal group, the MCI group displayed the following distinctions: higher smoking prevalence (p=0.008), a greater number of non-coffee drinkers (p<0.001), a lower percentage of individuals using dietary supplements (p=0.045), and more individuals with no exercise (p<0.001).

**Table 2:** Lifestyle of participants with/without MCI.

**Variable**	**Total (n=32,897)**	**MMSE^a^**		**p-value**
		**Normal (n=29,019)**	**MCI (n=3,878)**	
Smoking				0.008
None	26,459 (85.5%)	23,358 (85.7%)	3,101 (84.0%)	
Current/Former	4,497 (14.5%)	3,908 (14.3%)	589 (16.0%)	
Alcohol consumption				0.747
None	29,880 (90.9%)	26,364 (90.9%)	3,516 (90.7%)	
Current/Former	3,000 (9.1%)	2,641 (9.1%)	359 (9.3%)	
Tea consumption				0.28
No	8,345 (74.6%)	7,171 (74.8%)	1,174 (73.6%)	
Yes	2,834 (25.4%)	2,412 (25.2%)	422 (26.4%)	
Coffee consumption				<0.001
No	7,237 (64.7%)	6,101 (63.7%)	1,136 (71.2%)	
Yes	3,942 (35.3%)	3,483 (36.3%)	459 (28.8%)	
Dietary supplements consumption				0.045
None	4,325 (38.7%)	3,671 (38.3%)	654 (41.0%)	
Regular/Irregular	6,848 (61.3%)	5,906 (61.7%)	942 (59.0%)	
Meat intake preferences				0.605
Q1	2,792 (25.0%)	2,381 (24.9%)	411 (25.8%)	
Q2	2,792 (25.0%)	2,408 (25.1%)	384 (24.1%)	
Q3	2,792 (25.0%)	2,405 (25.1%)	387 (24.3%)	
Q4	2,792 (25.0%)	2,382 (24.9%)	410 (25.8%)	
Favor intake preferences				0.156
Q1	2,792 (25.0%)	2,364 (24.7%)	428 (26.9%)	
Q2	2,792 (25.0%)	2,385 (24.9%)	407 (25.6%)	
Q3	2,792 (25.0%)	2,409 (25.2%)	383 (24.1%)	
Q4	2,792 (25.0%)	2,418 (25.3%)	374 (23.5%)	
Dietary fat intake preferences				0.727
Q1	2,792 (25.0%)	2,381 (24.9%)	411 (25.8%)	
Q2	2,792 (25.0%)	2,409 (25.2%)	383 (24.1%)	
Q3	2,792 (25.0%)	2,398 (25.0%)	394 (24.7%)	
Q4	2,792 (25.0%)	2,388 (24.9%)	404 (25.4%)	
Exercise habits				<0.001
No	16,952 (51.6%)	14,818 (51.1%)	2,134 (55.0%)	
Yes	15,931 (48.4%)	14,188 (48.9%)	1,743 (45.0%)	
Physical Activity Accumulation				<0.001
None	16,952 (51.6%)	14,818 (51.1%)	2,134 (55.0%)	
≤20 METs/week	7,876 (24.0%)	7,052 (24.3%)	824 (21.3%)	
>20 METs/week	8,055 (24.5%)	7,136 (24.6%)	919 (23.7%)	
Physical Activity Intensity^b^				<0.001
Group 1	18,104 (55.1%)	15,849 (54.6%)	2,255 (58.2%)	
Group 2	12,945 (39.4%)	11,523 (39.7%)	1,422 (36.7%)	
Group 3	1,834 (5.6%)	1,634 (5.6%)	200 (5.2%)	

^a^MCI was defined as a score ≤18 for illiterates, ≤21 for participants with primary school education, and ≤25 for those with junior high school degree or above.

^b^Group1: no exercise habit or only conduct light-intensity physical activity (<3 MET/hr).

Group2: conduct physical activities including moderate-intensity physical activity (3-5.9 MET/hr).

Group3: physical activity including vigorous-intensity physical activity (≥6 MET/hr).

### Lifestyle risk factors for MCI

The lifestyle factors associated with MCI, as presented in Table 3, were analyzed after adjusting for age, gender, hypertension, diabetes, stroke, and hyperlipidemia. Smokers and former smokers exhibited a 1.19-fold higher risk of MCI (OR = 1.19, 95% CI: 1.07-1.33). Furthermore, participants who reported a habit of drinking coffee and taking dietary supplements demonstrated a lower risk of MCI. The risk of MCI was 28% lower for coffee drinkers (OR = 0.72, 95% CI: 0.64-0.80) and 12% lower for dietary supplement consumers (OR = 0.88, 95% CI: 0.79-0.98). Individuals who maintained a regular physical activity routine showed a 15% reduction in the risk of MCI (OR = 0.85, 95% CI: 0.80-0.91). Regardless of whether their weekly physical activity MET exceeded or was equal to 20 METs, participants experienced a decreased risk of MCI, with a 19% reduction in risk for those with METs/week ≤20 (OR = 0.81, 95% CI: 0.75-0.89) and a 11% reduction for those with METs/week >20 (OR = 0.89, 95% CI: 0.82-0.97). Regarding exercise types, individuals with physical activity including moderate-intensity physical activity had a 13% lower risk of MCI compared to those without exercise habit or only conduct light-intensity physical activity (OR = 0.87, 95% CI: 0.81-0.93). We additionally used two variables of physical activity (physical activity intensity levels and accumulation of physical activity per week) to conduct a cross-table analysis (Supplementary Table 3). In summary, participants with moderate-intensity physical activity and accumulation of metabolic equivalents of task ≤ 20 per week had a significantly lower risk of MCI (OR = 0.80, 95% CI: 0.73-0.88).

**Table 3:** Identification of lifestyle risk factors for MCI using logistic regression.

**Variable**	**Logistic regression, OR of MCI^a^ (95% CI)**		
	**Crude**	**Model 1^c^**	**Model 2^d^**
Smoking			
None	Ref.	Ref.	Ref.
Current/Former	1.14 (1.03 - 1.25)*	1.20 (1.08 - 1.34)*	1.19 (1.07 - 1.33)*
Alcohol consumption			
None	Ref.	Ref.	Ref.
Current/Former	1.02 (0.91 - 1.14)	1.04 (0.92 - 1.18)	1.03 (0.91 - 1.16)
Tea consumption			
No	Ref.	Ref.	Ref.
Yes	1.07 (0.95 - 1.21)	1.10 (0.97 - 1.24)	1.09 (0.96 - 1.23)
Coffee consumption			
No	Ref.	Ref.	Ref.
Yes	0.71 (0.63 - 0.79)*	0.72 (0.64 - 0.80)*	0.72 (0.64 - 0.80)*
Dietary supplements consumption			
None	Ref.	Ref.	Ref.
Regular/Irregular	0.90 (0.80 - 1.00)*	0.87 (0.78 - 0.97)*	0.88 (0.79 - 0.98)*
Meat intake preferences			
Q1	Ref.	Ref.	Ref.
Q2	0.92 (0.79 - 1.07)	0.92 (0.79 - 1.07)	0.92 (0.79 - 1.07)
Q3	0.93 (0.80 - 1.08)	0.92 (0.79 - 1.07)	0.92 (0.79 - 1.07)
Q4	1.00 (0.86 - 1.16)	0.99 (0.85 - 1.15)	0.99 (0.85 - 1.15)
Favor intake preferences			
Q1	Ref.	Ref.	Ref.
Q2	0.94 (0.81 - 1.09)	0.94 (0.81 - 1.09)	0.94 (0.81 - 1.09)
Q3	0.88 (0.76 - 1.02)	0.88 (0.76 - 1.02)	0.88 (0.76 - 1.03)
Q4	0.85 (0.74 - 0.99)*	0.87 (0.75 - 1.01)	0.87 (0.74 - 1.01)
Dietary fat intake preferences			
Q1	Ref.	Ref.	Ref.
Q2	0.92 (0.79 - 1.07)	0.93 (0.80 - 1.09)	0.94 (0.80 - 1.09)
Q3	0.95 (0.82 - 1.11)	0.96 (0.82 - 1.11)	0.96 (0.82 - 1.11)
Q4	0.98 (0.84 - 1.14)	1.00 (0.86 - 1.16)	1.00 (0.86 - 1.16)
Exercise habits			
No	Ref.	Ref.	Ref.
Yes	0.85 (0.80 - 0.91)*	0.84 (0.79 - 0.90)*	0.85 (0.80 - 0.91)*
Physical Activity Accumulation			
None	Ref.	Ref.	Ref.
≤20 METs/week	0.81 (0.75 - 0.88)*	0.80 (0.74 - 0.87)*	0.81 (0.75 - 0.89)*
>20 METs/week	0.89 (0.82 - 0.97)*	0.88 (0.81 - 0.96)*	0.89 (0.82 - 0.97)*
Physical Activity Intensity^b^			
Group 1	Ref.	Ref.	Ref.
Group 2	0.87 (0.81 - 0.93)*	0.86 (0.80 - 0.92)*	0.87 (0.81 - 0.93)*
Group 3	0.86 (0.74 - 1.00)	0.87 (0.74 - 1.01)	0.88 (0.75 - 1.03)

^a^MCI was defined as a score ≤18 for illiterates, ≤21 for participants with primary school education, and ≤25 for those with junior high school degree or above.

^b^Group1: no exercise habit or only conduct light-intensity physical activity (<3 MET/hr).

Group2: conduct physical activities including moderate-intensity physical activity (3-5.9 MET/hr).

Group3: physical activity including vigorous-intensity physical activity (≥6 MET/hr).

^c^Model 1: adjusted by age, gender.

^d^Model 2: adjusted by age, gender, hypertension, diabetes, stroke, hyperlipidemia.

*p<0.05.

### Lifestyle risk factors stratified by gender for MCI

The lifestyle factors affecting MCI in different genders are presented in Table 4 after adjusting for age, hypertension, diabetes, stroke, and hyperlipidemia. Among males, smokers had a 1.2-fold higher risk of MCI (OR = 1.2, 95% CI: 1.06-1.34), while this association was not observed in females (OR = 1.16, 95% CI: 0.85-1.59). Diverse flavored intake pattern (Q4) in female, compared to Q1, was associated with cognitive protection (OR = 0.8, 95% CI: 0.66-0.97). Additionally, in terms of physical activity accumulation, METs/week >20 in male (OR = 0.8, 95% CI: 0.70-0.92) and METs/week ≤20 in female (OR = 0.78, 95% CI: 0.70-0.87) acted as protective factors for MCI.

**Table 4:** Identification of lifestyle risk factors stratified by gender for MCI using logistic regression.

**Variable**	**Logistic regression, OR of MCI^a^ (95% CI)**					
	**Male(n=12,862)**			**Female(n=20,035)**		
	**Crude**	**Model 1^c^**	**Model 2^d^**	**Crude**	**Model 1^c^**	**Model 2^d^**
Smoking						
None	Ref.	Ref.	Ref.	Ref.	Ref.	Ref.
Current/Former	1.19 (1.06 - 1.33)*	1.21 (1.08 - 1.36)*	1.20(1.06 - 1.34)*	1.14 (0.84 - 1.56)	1.17 (0.86 - 1.61)	1.16 (0.85 - 1.59)
Alcohol consumption						
None	Ref.	Ref.	Ref.	Ref.	Ref.	Ref.
Current/Former	1.02 (0.89 - 1.17)	1.03 (0.90 - 1.18)	1.01 (0.89 - 1.16)	1.10 (0.82 - 1.47)	1.11 (0.83 - 1.48)	1.10 (0.82 - 1.48)
Tea consumption						
No	Ref.	Ref.	Ref.	Ref.	Ref.	Ref.
Yes	1.15 (0.97 - 1.38)	1.17 (0.98 - 1.40)	1.16 (0.97 - 1.38)	1.03 (0.86 - 1.22)	1.04 (0.87 - 1.23)	1.03 (0.87 - 1.23)
Coffee consumption						
No	Ref.	Ref.	Ref.	Ref.	Ref.	Ref.
Yes	0.72 (0.60 - 0.86)*	0.73 (0.60 - 0.87)*	0.73 (0.61 - 0.88)*	0.70 (0.60 - 0.82)*	0.71 (0.61 - 0.82)*	0.71 (0.61 - 0.82)*
Dietary supplements consumption						
None	Ref.	Ref.	Ref.	Ref.	Ref.	Ref.
Regular/Irregular	0.89 (0.75 - 1.05)	0.88 (0.74 - 1.04)	0.89 (0.75 - 1.05)	0.88 (0.76 - 1.01)	0.87 (0.75 - 1.00)	0.87 (0.76 - 1.01)
Meat intake preferences						
Q1	Ref.	Ref.	Ref.	Ref.	Ref.	Ref.
Q2	0.85 (0.68 - 1.07)	0.85 (0.68 - 1.07)	0.84 (0.67 - 1.06)	0.98 (0.80 - 1.20)	0.98 (0.80 - 1.20)	0.98 (0.80 - 1.21)
Q3	0.95 (0.75 - 1.20)	0.94 (0.74 - 1.19)	0.94 (0.75 - 1.19)	0.92 (0.75 - 1.12)	0.92 (0.75 - 1.12)	0.92 (0.75 - 1.12)
Q4	0.93 (0.73 - 1.19)	0.93 (0.72 - 1.19)	0.93 (0.72 - 1.19)	1.02 (0.84 - 1.24)	1.03 (0.85 - 1.24)	1.03 (0.85 - 1.25)
Favor intake preferences						
Q1	Ref.	Ref.	Ref.	Ref.	Ref.	Ref.
Q2	0.99 (0.77 - 1.27)	0.99 (0.77 - 1.28)	1.00 (0.77 - 1.28)	0.92 (0.77 - 1.11)	0.92 (0.76 - 1.10)	0.91 (0.76 - 1.10)
Q3	0.95 (0.74 - 1.22)	0.96 (0.75 - 1.23)	0.96 (0.75 - 1.23)	0.85 (0.70 - 1.02)	0.84 (0.70 - 1.02)	0.84 (0.70 - 1.02)
Q4	0.97 (0.76 - 1.23)	0.98 (0.77 - 1.25)	0.98 (0.77 - 1.25)	0.79 (0.65 - 0.96)*	0.80 (0.66 - 0.97)*	0.80 (0.66 - 0.97)*
Dietary fat intake preferences						
Q1	Ref.	Ref.	Ref.	Ref.	Ref.	Ref.
Q2	0.83 (0.63 - 1.08)	0.83 (0.63 - 1.08)	0.83 (0.63 - 1.08)	0.97 (0.81 - 1.17)	0.99 (0.82 - 1.18)	0.99 (0.82 - 1.18)
Q3	0.86 (0.67 - 1.11)	0.85 (0.66 - 1.10)	0.86 (0.66 - 1.11)	1.01 (0.84 - 1.22)	1.02 (0.84 - 1.22)	1.01 (0.84 - 1.22)
Q4	0.95 (0.74 - 1.21)	0.95 (0.74 - 1.21)	0.95 (0.74 - 1.21)	1.00 (0.82 - 1.21)	1.01 (0.83 - 1.22)	1.00 (0.82 - 1.22)
Exercise habits						
No	Ref.	Ref.	Ref.	Ref.	Ref.	Ref.
Yes	0.84 (0.75 - 0.93)*	0.83 (0.74 - 0.92)*	0.84 (0.75 - 0.93)*	0.86 (0.79 - 0.94)*	0.85 (0.78 - 0.93)*	0.87 (0.80 - 0.95)*
Physical Activity Accumulation						
None	Ref.	Ref.	Ref.	Ref.	Ref.	Ref.
≤20 METs/week	0.87 (0.76 - 1.00)	0.87 (0.75 - 1.00)	0.88 (0.76 - 1.01)	0.78 (0.70 - 0.87)*	0.77 (0.69 - 0.86)*	0.78 (0.70 - 0.87)*
>20 METs/week	0.81 (0.71 - 0.92)*	0.79 (0.69 - 0.90)*	0.80 (0.70 - 0.92)*	0.96 (0.87 - 1.07)	0.95 (0.86 - 1.06)	0.97 (0.87 - 1.07)
Physical Activity Intensity^b^						
Group 1	Ref.	Ref.	Ref.	Ref.	Ref.	Ref.
Group 2	0.83 (0.74 - 0.94)*	0.82 (0.73 - 0.92)*	0.83 (0.74 - 0.93)*	0.89 (0.81 - 0.97)*	0.88 (0.80 - 0.96)*	0.89 (0.81 - 0.97)
Group 3	0.82 (0.67 - 1.00)*	0.82 (0.67 - 1.00)	0.83 (0.68 - 1.01)	0.93 (0.73 - 1.20)	0.94 (0.73 - 1.20)	0.96 (0.75 - 1.23)

^a^MCI was defined as a score ≤18 for illiterates, ≤21 for participants with primary school education, and ≤25 for those with junior high school degree or above.

^b^Group 1: no exercise habit or only conduct light-intensity physical activity (<3 MET/hr).

Group 2: conduct physical activities including moderate-intensity physical activity (3-5.9 MET/hr).

Group 3: physical activity including vigorous-intensity physical activity (≥6 MET/hr).

^c^Model 1: adjusted by age.

^d^Model 2: adjusted by age, hypertension, diabetes, stroke, hyperlipidemia.

*p<0.05.

## DISCUSSION

This study reveals that the prevalence of MCI among individuals aged 60 and above is 11.78%. It demonstrates the role of former or current smoking as a risk factor for MCI. In contrast, maintaining a regular exercise routine, consuming coffee, and using dietary supplements are identified as protective factors for MCI. Furthermore, the research highlights the gender-specific differences in lifestyle factors and their impact on cognitive function. For males, engaging in high-intensity physical activity is beneficial for cognitive function, implying that males may benefit from more intense or longer-duration physical activities to reduce their risk of MCI. On the other hand, for females, moderate physical activity and a diverse flavored intake pattern appear to be more supportive of cognitive function.

The prevalence of MCI increases significantly with advancing age [19]. Article conducted in Taiwan reveals a higher prevalence among females compared to males [20], whereas research in China indicates a steeper rise in prevalence with age among females compared to males [21]. In the current study, the prevalence ratios of males and females in the young-old group were similar (male 11.67%, female 11.86%), and consistent with prior research findings.

Numerous studies have indicated that higher levels of physical activity contribute to improved learning and memory [22, 23]. Epidemiological research suggests that regular physical activity can reduce the risk of cognitive decline in older adults [24, 25]. Our study also verifies this finding. Considering that physical activity can alter neural plasticity, promote the maintenance and growth of neurons, and influence synaptic connectivity [26], this emphasizes the importance of maintaining regular exercise with advancing age.

Notably, in addition to the influence of exercise routines, our findings also underline gender disparities in weekly exercise MET levels. Higher levels of physical activity METs per week in males and moderate levels in females were identified as protective factors in MCI. Previous study reported that different exercise interventions influence cognition and brain health in older adults and these effects may be gender dependent [27]. The biological mechanisms underlying the beneficial effects of exercise on the brain may be different in males and females, such as brain-derived neurotrophic factor (BDNF) is a critical mediator of the beneficial effect of exercise on brain function [28, 29], BDNF facilitates cognition performance through modulation of neurotransmission. However, exercise increased levels of BDNF in females but decreased levels in males [30]. This may explain the divergent contribution of physical activity to the phenotype of MCI in male and female.

Previous study showed that high dietary diversity was associated with a lower proportion of MCI [31], which is consistent with our finding in female. Females prefer to have diverse tastes may positively stimulate neural feedback to make it more beneficial in neuroplasticity [32]. Moreover, the diversity of food selection implies the intake of more varied and sufficient nutrients, potentially exerting a protective effect against brain aging through neural stimulation. The potential mechanisms of the present findings might involve the complex effects of various nutrients on cognitive function due to diet diversity. First, a previous study reported that calcium and magnesium reduce the risk of developing dementia [33]. Second, the intake of amino acids, especially lysine, phenylalanine, threonine, and alanine, is positively associated with cognitive function in late life [34]. Third, a large prospective cohort study showed that a high consumption of fish was associated with a lower risk of dementia [35]. However, there is no significant association between flavor diet and MCI in male, which may be due to the gender differences in taste and food habits. Future studies should validate the gender difference mentioned in this study.

A community study in the US suggested that current smokers had 1.33 times (95% CI: 1.12-1.59) the risk of all-cause dementia compared to never smokers, while those who quit smoking in the last 9 years had 1.24 times (95% CI: 1.01-1.52) the risk of all-cause dementia compared to never smokers and quitting for more than 9 years had no association with dementia [36]. Other articles have also found that smokers and former smokers had an increased risk of MCI [37], consistent with the results of our study.

In the context of coffee consumption habits, the Italian longitudinal study on aging has shown that elderly individuals who habitually consume moderate amounts of coffee (1-2 cups per day) have a lower risk of developing MCI compared to those who never or rarely (1 cup per day) consume coffee [38]. This finding aligns with the results of our study and highlights the potential benefits of habitual coffee intake on cognitive function.

Previous research has already established that the intake of antioxidant and anti-inflammatory nutrients such as Omega-3 fatty acids, curcumin, flavonoids, vitamin B12, vitamin D, vitamin E, choline, complex B vitamins, and iron can help combat cognitive decline [39]. Additionally, previous study also suggests that supplementation with vitamin E, magnesium, ginkgo biloba, green tea polyphenols, phospholipids, and polyunsaturated fatty acids (e.g., DHA, EPA) can effectively reduce the incidence of MCI [40]. While our study couldn’t specify which dietary supplements are beneficial for cognitive function, the results align with the notion that dietary supplements can have a positive impact on cognitive function. Future studies should analyze the detailed components of supplements to reveal the detailed mechanism.

This study has several limitations. Firstly, this study is a cross-sectional study, which could not explore causal relationships. Secondly, this study relies on self-reported dietary habits of the participants. This approach does not provide specific details about the frequency and quantity of their diet, and dietary characteristics might be subject to personal interpretation. Thirdly, the study population consists of volunteers who participated in the TWB, which might lead to a selection bias. These individuals may have better health awareness and healthier lifestyles, potentially resulting in lower risk profiles compared to the general elderly population.

## CONCLUSIONS

This study reveals that individuals who consume coffee, take dietary supplements, and maintain regular physical activity habits exhibit better cognitive function. It is worth noting that male is advised to target a higher physical activity metabolic equivalent, while female can attain cognitive benefits with moderate physical activity. Moreover, diversified dietary preferences among females but not males serve as protective factors for cognitive function. In the future, it is recommended to conduct longitudinal studies to explore causal relationships in this regard.

## Supplementary Material

Supplementary Tables
