# Supplementary material for: Gender-specification lifestyle factors associated with mild cognitive impairment among young-old adults in Taiwan
Source: Aging (Albany NY). 2024 Dec 10;16(22):13662–75. doi: 10.18632/aging.206172 (PMC11723654; doi:10.18632/aging.206172)
Supplement: Supplementary Tables [file aging-16-206172-s001.pdf]

SUPPLEMENTARY TABLES

Supplementary Table 1. The dietary patterns were derived from structured questionnaire through principal components analysis.

Rotation component matrix[a]			
	Dietary ingredients		
	Meat	Flavor	Fats
Do you choose to eat lean meat instead of pork belly or pork knuckle?	.720		
Do you try to eat meat with lower fat content such as fish or chicken instead of meat with higher fat content such as pork or beef?	.704		
When you eat meat, do you choose to eat meat that is cooked in a braised or roasted way (e.g., braised or roasted chicken drumsticks) instead of fried meat (e.g., fried chicken cutlets)?	.662		
Do you eat poultry/meat as fewer as possible?	.656		
Do you add salt, soy sauce, soy paste, pepper salt, chili sauce or other seasonings to your meals?		.652	
Do you accompany your meals with pickled vegetables, fermented tofu and fermented soybeans?		.601	
Do you eat noodles/rice with lard or fried vegetables?		.583	
Do you fry bean products (tofu, bean curd) before eating?		.548	
Do you spread cream, butter, or mayonnaise on bread before eating?		.536	
Do you cook meat/fish with oil?			.732
Do you eat meat together with the skin?			.666
Do you fry vegetables before eating?			.610

Extraction method: Principal component analysis.
Rotation method: Using Kaiser's normal varimax.
[a]: Rotation to converge in 4 iterations.
The responses to these questions were presented on a five-point scale (1 = never, 2 = seldom, 3 = sometimes, 4 = frequently, and 5 = always).

Supplementary Table 2. Various types of physical activity and metabolic equivalent of tasks (METs).

Walking	3.3 METs	Wai-Tan-Kung	3.8 METs	Badminton	5.2 METs	Aerobic dance	5.0 METs
Jogging	3.8 METs	Neidan	3.8 METs	Table tennis	4.9 METs	Folk Dance	4.5 METs
Brisk walking	5.0 METs	Falun Gong	3.8 METs	Foot ball	9.0 METs	Bicycling	6.0 METs
Rope skipping	8.0 METs	Yuan Chih Dance	3.8 METs	Golf	3.8 METs	Hiking	4.0 METs
Swimming	4.0 METs	Tai Chi	3.8 METs	Croquet	2.5 METs	Strength training	4.5 METs
Gymnastics	4.0 METs	Xiang Gong	3.8 METs	Tennis	7.0 METs	Climbing stairs	5.0 METs
Swing hands exercise	2.5 METs	Yoga	2.5 METs	Basketball	6.0 METs	Hula hoop	3.5 METs

The metabolic equivalent of task (MET) was calculated with reference to the study by Hiraike et al. [16].

Supplementary Table 3. The cross-table analysis of physical activity intensity levels and accumulation for MCI.

Physical activity accumulation		Physical activity intensity N (%), OR (95% CI)		
		**Group1**	**Group 2**	**Group 3**
	> 20 METs/week	N = 47 (0.1%)<br>OR = 1.02 (0.43 - 2.40)	N = 6,671 (20.3%)<br>OR = 0.91 (0.83 - 0.99)*	N = 1,337 (4.1%)<br>OR = 0.82 (0.68 - 0.98)*
	≤ 20 METs/week	N = 1,105 (3.4%)<br>OR = 0.81 (0.66 - 0.98)*	N = 6,274 (19.1%)<br>OR = 0.80 (0.73 - 0.88)*	N = 497 (1.5%)<br>OR = 0.94 (0.71 - 1.23)
	None	N = 16,952 (51.6%)<br>Ref.	NA	NA

[a]Group 1: no exercise habit or only conduct light-intensity physical activity (<3 MET/hr).
Group 2: conduct physical activities including moderate-intensity physical activity (3-5.9 MET/hr).
Group 3: conduct physical activity including vigorous-intensity physical activity (≥6 MET/hr).
*p < 0.05.
